# Impact of long-term elosulfase alfa treatment on clinical and patient-reported outcomes in patients with mucopolysaccharidosis type IVA: results from a Managed Access Agreement in England

**DOI:** 10.1186/s13023-021-01675-x

**Published:** 2021-01-21

**Authors:** Maureen Cleary, James Davison, Rachel Gould, Tarekegn Geberhiwot, Derralynn Hughes, Jean Mercer, Alexandra Morrison, Elaine Murphy, Saikat Santra, James Jarrett, Swati Mukherjee, Karolina M. Stepien

**Affiliations:** 1 Department of Metabolic Medicine, Great Ormond Street Hospital, Great Ormond St., London, WC1N 3JH UK; 2 NIHR Biomedical Research Centre London, London, UK; 3 Birmingham Women’s and Children’s NHS Foundation Trust, Birmingham, UK; 4 University Hospital Birmingham, Birmingham, UK; 5 Royal Free NHS Foundation Trust and University College London, London, UK; 6 Saint Mary’s Hospital, Manchester, UK; 7 Rare Disease Research Partners, Amersham, UK; 8 National Hospital for Neurology and Neurosurgery, London, UK; 9 BioMarin International Ltd., London, UK; 10 Salford Royal NHS Foundation Trust, Salford, UK

**Keywords:** Clinical outcomes, Elosulfase alfa, Enzyme replacement therapy, Managed access agreement, Mucopolysaccharidosis IVA, Patient-reported outcomes

## Abstract

**Background:**

We present baseline characteristics and follow-up data of a Managed Access Agreement (MAA), including patients with mucopolysaccharidosis IVA (MPS IVA) receiving elosulfase alfa enzyme replacement therapy (ERT) in England on a conditional basis. Patients enrolled in the MAA programme are reviewed on an annual basis. Therapy can be continued if patients are compliant, able to tolerate infusions, and meet four out of five pre-defined clinical and patient-reported outcomes (PRO) criteria. Baseline and follow-up clinical and PRO data are presented for all participants who completed ≥ 1 year of assessments in the MAA.

**Results:**

The analysis included data from 55 patients, including 26 patients previously enrolled in clinical trials and 29 who started ERT after enrolling in the MAA. In patients with both baseline and follow-up data, mean 6-min walk test distance increased from 217 m at baseline to 244 m after a mean follow-up of 4.9 years. Improvement or stabilisation was seen regardless of age at treatment initiation or duration of treatment. Mean forced vital capacity and forced expiratory volume in 1 s were 0.87 L and 0.78 L, respectively at baseline and 1.05 L and 0.88 L after a mean follow-up of 5.5 years. PRO data showed overall improvements over time in Mobility, Self-care, and Caregiver assistance scores of the MPS-Health Assessment Questionnaire, relatively stable quality of life, and some improvements in pain scores.

**Conclusions:**

The MAA data confirm the effects of elosulfase alfa on clinical and PRO results observed in the clinical trials and provide real-world evidence for long-term stabilisation in these measures, suggesting a positive impact on the natural history of MPS IVA.

## Background

Mucopolysaccharidosis IVA (MPS IVA), or Morquio A syndrome, is an ultra-rare, multi-systemic disorder caused by a deficiency of the enzyme *N*-acetylgalactosamine-6-sulfatase (GALNS; EC 3.1.6.4). GALNS deficiency results in impaired catabolism of the glycosaminoglycans (GAGs) keratan sulfate (KS) and chondroitin-6-sulfate and lysosomal accumulation of the non-degraded GAGs throughout the body [1].

Patients with MPS IVA typically appear healthy at birth, but gradually develop multi-systemic manifestations that progress over time [2]. Affected patients show a wide genotypic and phenotypic heterogeneity, but life-threatening symptoms ultimately develop in all phenotypes, resulting in premature mortality [1, 3]. Characteristic features of the disease include short stature, skeletal and joint abnormalities, cardiopulmonary dysfunction, impaired vision (corneal clouding, glaucoma), hearing loss, spinal cord compression and hepatomegaly [4, 5]. The combination of respiratory and cardiac dysfunction, musculoskeletal impairment and short stature associated with MPS IVA results in severely impaired endurance and pulmonary function, in turn affecting the patients’ ability to perform daily activities and their quality of life (QoL) [1, 6, 7]. Natural history data from the Morquio A Clinical Assessment Program (MOR-001) demonstrated that patients show a decline in endurance over time [8]. Pulmonary function measures (forced vital capacity [FVC] and maximum voluntary ventilation) increased in patients aged ≤ 14 years, likely due to growth, but decreased over time in older patients [8].

International management guidelines for MPS IVA recommend elosulfase alfa as a first-line treatment in conjunction with a multidisciplinary management approach [9]. Elosulfase alfa is an enzyme replacement therapy (ERT) which targets the underlying pathology of the disease and was approved by the European Medicines Agency in April 2014 [10]. Its efficacy and safety have been demonstrated in a major clinical research programme [11–16]. The pivotal double-blind, placebo-controlled, phase 3 study (MOR-004, N = 176) showed a statistically significant improvement in 6-min walk test (6MWT) distance versus placebo and a rapid and sustained decrease in urinary KS (uKS) over 24 weeks in patients receiving elosulfase alfa. The long-term extension of the study (MOR-005) showed durability of the 6MWT and uKS improvements over 120 weeks, as well as sustained numerical improvements in pulmonary function measures and activities of daily living (ADL) assessed with the MPS Health Assessment Questionnaire (MPS-HAQ) [11, 13, 14, 17]. The long-term MOR-004/005 results were significantly better than those of untreated patients from the MOR-001 study over a similar period of time, suggesting at least a slower decline in endurance and pulmonary function and partial recovery of functional abilities with ERT [13, 14, 17].

Elosulfase alfa was considered well tolerated, with an acceptable safety profile. Most drug-related adverse events (AEs) were mild or moderate infusion-associated reactions that could be managed easily [11, 13]. In addition, a smaller open-label, single-arm phase 1/2 study, MOR-002 (N = 20) and its long-term extension MOR-100 (N = 17) showed maintained tolerability and a favourable safety profile as well as stable endurance, respiratory function, and ability to perform ADL over 5 years [18].

More long-term efficacy and safety data in a broader patient population are being collected in the Morquio A Registry Study (MARS; clinicaltrials.gov NCT02294877), an on-going multicentre, multinational, observational disease registry for patients diagnosed with MPS IVA.

The present paper summarises clinical and patient-reported outcomes (PRO) from MPS IVA patients receiving ERT in England. Since December 2015, patients in England with MPS IVA are granted access to treatment with elosulfase alfa on a conditional basis through a managed access agreement (MAA) [19]. This agreement is a collaboration between the National Health Service (NHS) England, the National Institute for Health and Care Excellence (NICE), the treating physicians, the UK MPS Society, and the manufacturer (BioMarin). To continue treatment, patients must show improvements or stabilisation of disease as measured by clinical outcomes, laboratory markers, and PRO tools.

## Methods

### Design and objectives of the MAA

The MAA was initiated in December 2015 and will continue for a maximum of 5 years. Patients in England are eligible for treatment with elosulfase alfa 2.0 mg/kg/week if they meet specific starting criteria [19], as specified in Additional file 1: Table S1. Patients aged ≥ 5 years can only start treatment once a full set of baseline assessments is obtained. All patients or their parents provided written, signed informed consent to participate in the programme.

As part of the MAA programme, patients are monitored regularly with annual review of assessment results (see overview of assessments in Additional file 1: Table S2). Patients have to cease therapy if they are non-compliant (defined as missing ≥ 3 infusions in any 14 month period without medical reasons), are unable to tolerate infusions due to infusion-related reactions that cannot be controlled, or fail to meet four of the five criteria outlined in Table 1. These criteria were based largely on clinical trial outcomes, but agreed upon by a group of clinical experts and commissioners.

**Table 1:** MAA criteria for maintaining treatment

**Clinical criteria** Treatment-naïve patients: criteria following the first year of treatment • Improvement in 6MWT distance or the timed 25-foot (7.6 m) walk (T25FW) of ≥ 10% over baseline^a^ or stabilisation after 10% improvement^b^ • Improvement in FVC or FEV_1_ of ≥ 5% over baseline or stabilisation after 1 year • Decline in LVEF of < 10% from baseline • Decline of uKS of ≥ 20% from baseline (and stabilised)
Ex-trial patients or patients receiving treatment for over 12 months • 6MWT or T25FW remains ≥ 5% above the baseline value at the start of treatment • FVC and FEV_1_ remain ≥ 2% above the baseline value at the start of treatment • uKS levels remain reduced ≥ 20% from baseline • Decline in LVEF of < 10% from baseline
**PRO criteria** (same for treatment-naïve and ex-trial patients): • No adverse change in numerical value^c^ of two out of three of the following: • EQ-5D-5L score OR MPS-HAQ Caregiver Burden score • Beck Depression Score (≥ 13 years) • APPT/BPI pain severity score (depending on age)

*6MWT* 6-min walk test, *APPT* adolescent pediatric pain tool, *BPI* Brief Pain Inventory, *EQ-5D-5L* EuroQol 5 dimensions, 5 levels, *FEV*_*1*_ forced expiratory volume in 1 s, *FVC* forced vital capacity, *LVEF* left ventricular ejection fraction, *MPS-HAQ* MPS Health Assessment Questionnaire, *PRO* patient-reported outcome, *uKS* urinary keratan sulphate

^a^As measured at the annual assessment

^b^6MWT distance initially increased by 10% or more versus baseline, and then remained at least 5% above the baseline value at the start of treatment

^c^The MAA agreement did not specify how adverse change was defined for each instrument. Patients were assessed on a case-by-case basis where no adverse change means absolute scores remain stable (within the same category) or improve

Given the progressive nature of MPS IVA, patients receiving treatment in clinical trials prior to the initiation of the MAA (ex-trial patients) and those receiving treatment for over 12 months would be expected to have declined in function relative to baseline further than patients initiating treatment in the MAA (treatment-naïve patients) during the first year of treatment. Therefore, these two groups are evaluated based on different criteria in the MAA (Table 1) and were analysed separately.

The key objectives of the present analysis were to present baseline characteristics and follow-up data of the MAA (data-cut May 2019) and to compare these data with those of untreated patients from the MOR-001 natural history study.

### Evaluation of clinical and patient-reported outcomes

Clinical outcomes discussed here include uKS, weight, 6MWT results, pulmonary function (FVC and forced expiratory volume in 1 s [FEV_1_]) and LVEF. LVEF was reported as part of the standard clinically acquired echocardiographic examination. It was calculated by dividing the stroke volume (SV) by the end diastolic volume (EDV): LVEF = (SV/EDV) × 100. PRO measures include ADL, QoL, depression (for those aged ≥ 13 years), and pain.

ADL were monitored using the MPS-HAQ. The MPS-HAQ assesses self-care (eating/drinking, dressing, bathing, grooming, tooth brushing, and toileting), mobility skills (dexterity, mobility, walking, stair climbing, and gross motor skills), and caregiver-assistance required in the performance of these activities [17]. Total self-care and mobility domain scores range from 0 (not difficult at all) to 10 (extremely difficult) and 11 (unable to do). The total caregiver-assistance domain score ranges from 13 (independent) to 52 (complete assistance required) [17]. Decreases in MPS-HAQ scores imply improvements.

QoL was monitored using the EuroQol 5 dimensions, 5 levels (EQ-5D-5L) tool, a generic standardised measure of health status comprising five dimensions: Mobility, Self-care, Usual activities, Pain/Discomfort and Anxiety/Depression [20]. EQ-5D-5L health states can be converted into a single summary index value (utility), ranging from “1” (representing perfect health) to “0” (representing death). The summary index was calculated using the EQ-5D value set for the UK [20].

Pain was measured using patient-reported questionnaires, i.e. the Adolescent and Paediatric Pain Tool (APPT) in patients < 18 years of age and the Brief Pain Inventory (BPI) in patients aged ≥ 18 years. The APPT is a validated tool to evaluate pain severity, location and description in children and adolescents aged 8 to 17 years [21]. The BPI Short Form (BPI-SF) is a widely used tool to rate pain severity, pain location and the impact of pain on daily functioning [22]. Pain severity scores derived from the BPI were based on question 5 (Please rate your pain by telling me the one number that best describes your pain on the average), with scores ranging from 0 (no pain) to 10 (worst possible pain/pain as bad as you can imagine). In the APPT, pain severity is scored on the Word Graphic Rating Scale as 0 (no pain), 2 (little pain), 4 (medium pain), 6 (large pain), and 8 (worst possible pain).

Depression was monitored in patients aged ≥ 13 years using the Beck Depression Inventory (BDI), a 21-item self-report instrument, with higher total scores (ranging from 0 to 63) indicating more severe depressive symptoms [23].

PRO tools were completed on entry in the MAA and at least once before or at 12 months (see assessment schedule in Additional file 1: Table S2). PRO tools were completed by either the patient or their parent/caregiver (depending on the patient’s age) either over the telephone or during a face-to-face interview with a professional researcher.

For each clinical outcome, patients were measured against their pre-treatment baseline, if available. For those patients who did not have a pre-treatment baseline due to age, or when the variable was not measured at baseline for ex-trial patients, the first measure during the MAA period was used as baseline. Although most of the trials included the clinical outcomes discussed, they did not consistently contain the PROs. Therefore, with exception of the MPS-HAQ, all PRO baselines were measured when patients entered the MAA.

### Safety evaluation

The MAA does not track safety information, except monitoring intolerance to treatment and antibody titres.

### Statistical analysis

All statistical analyses were performed using SAS software. Baseline demographics and characteristics were summarised for all participants who completed ≥ 1 year of assessments. Results are presented separately for ex-trial patients and patients who were treatment-naïve when entering the MAA, as well as for all participants combined. The rate of decline in endurance (6MWT), lung function (FVC and FEV_1_), and ADL (MPS-HAQ) in untreated MPS IVA patients has been published previously [8] and is presented together with the MAA results to put them in context of the natural history of the disease. Descriptive statistics were summarised for absolute values over time and for the actual and percent change for each measure from baseline. A two-sample *t*-test was used to compare the means at baseline and last follow-up among patients completing both assessments.

Subgroup analyses were performed by age at treatment initiation (< 18 or ≥ 18 years) and for the patients from the first-in-human trial (MOR-002) who have been receiving treatment for longer than any other patient group, making their results of particular interest.

## Results

### Patient disposition and baseline characteristics

As of May 2019, 66 patients were enrolled in the MAA. Six patients stopped treatment during the study period: five voluntarily stopped for a variety of reasons (one patient left the country, four were unwilling to adhere to the treatment schedule or had a perceived lack of benefit) and one stopped due to failure to meet the MAA criteria to remain on treatment. Another five patients did not have follow-up data due to starting within a year of the analysis. Infusion data were available for 50 patients. The mean number of missed infusions was 0.93 in year 1 (N = 43), 0.64 in year 2 (N = 25), and 1.19 in year 3 (N = 27). All missed infusions were due to holiday or for medical reasons (intravenous access issues, hospitalisation, illness). One patient missed four infusions (including three that were medically approved) in year 3. All other patients missed ≤ 3 infusions yearly.

Of the 55 patients included in the analysis (Additional file 1: Figure S1), 26 patients started elosulfase alfa in clinical trials prior to enrolling in the MAA (ex-trial patients); the remaining 29 patients started elosulfase alfa after enrolling in the MAA (Table 2). Among the ex-trial patients, three (12%) were below 5 years of age. In the treatment-naïve group, eight patients (28%) were below 5 years of age.

**Table 2:** Patient demographics and baseline characteristics

	Ex-trial patients	Treatment-naïve patients	All patients
N	26	29	55
Female, number (%)	13 (50.0%)	15 (51.7%)	28 (50.9%)
Male, number (%)	13 (50.0%)	14 (48.3%)	27 (49.1%)
Age at enrolment, years			
N	26	29	55
Mean (SD)	14.4 (11.43)	15.3 (15.30)	14.9 (13.50)
Median	9	8	9
Min, max	4, 41	2, 58	2, 58
Weight (kg)			
N	26	25	51
Mean (SD)	28.0 (13.16)	25.04 (15.72)	26.6 (14.58)
Median	23.8	16.5	21.7
Min, max	14.2, 65.0	10.3, 62.6	10.3, 65.0
Treatment duration, years*			
N	26	29	55
Mean (SD)	7.46 (1.62)	2.36 (0.88)	4.78 (2.86)
Median	6.96	2.98	3.00
Min, max	4.08, 9.54	0.99, 3.04	0.99, 9.54
6MWT, m			
N	23	24	47
Mean (SD)	220.1 (91.47)	193.5 (105.80)	206.7 (98.86)
Median	228	184.5	215
Min, max	60, 433	20, 420	20, 433
FVC, L			
N	25	18	43
Mean (SD)	0.9 (0.66)	1.2 (0.97)	1.0 (0.80)
Median	0.6	0.8	0.7
Min, max	0.3, 2.9	0.3, 3.9	0.3, 3.9
FEV_1_, L			
N	25	18	43
Mean (SD)	0.8 (0.55)	0.9 (0.69)	0.8 (0.61)
Median	0.6	0.7	0.6
Min, max	0.2, 2.5	0.3, 2.4	0.2, 2.5
LVEF, %			
N	21	22	43
Mean (SD)	66 (6)	66 (10)	65 (8.2)
Median	65	69	65
Min, max	55, 80	36, 82	36, 82
uKS, µg/mg creatinine			
N	26	25	51
Mean (SD)	28.0 (16.01)	37.5 (23.3)	32.7 (20.27)
Median	32.4	44.3	35.1
Min, max	3.5, 50.3	2.6, 74.7	2.6, 74.7

^*^Treatment duration at last follow-up

*FVC* forced vital capacity, *FEV*_*1*_ forced expiratory volume in one second, *kg* kilograms, *LVEF* left ventricular ejection fraction, *N* number of patients, *SD* standard deviation, *uKS* urine keratan sulphate

### Clinical outcomes

#### Urinary keratan sulfate (uKS)

**Fig. 1:**
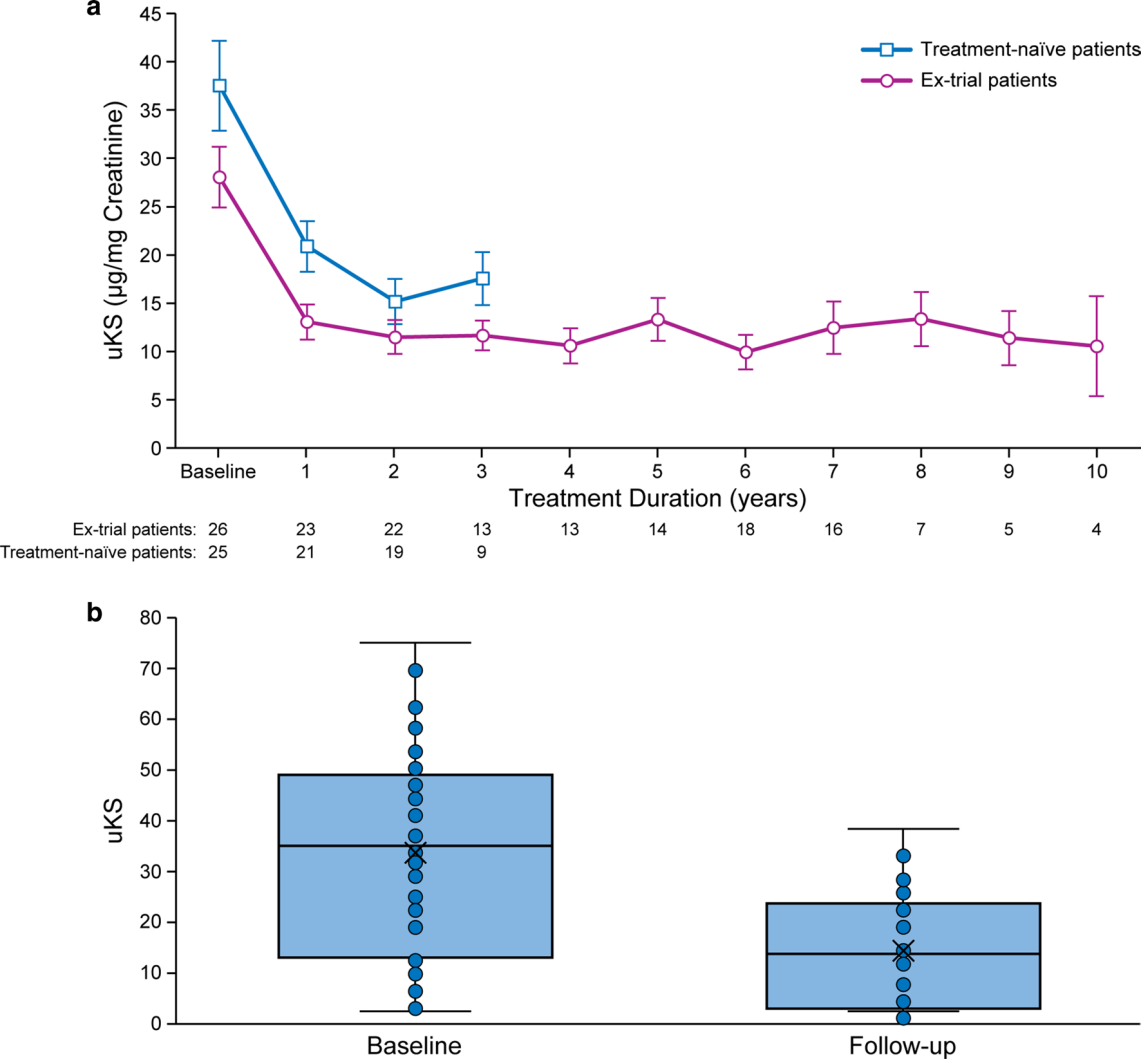
Mean urinary keratan sulfate (uKS) over time (**a**) and box plot comparing uKS at pre-treatment baseline and at last follow-up among patients with both measures (N = 48; mean follow-up 4.9 years) (**b**). In the box plot, lines show medians and first and third quartiles; asterisks show means

Mean uKS decreased rapidly and remained stable over time thereafter, regardless of treatment duration (Fig. 1a). In patients with both pre-treatment baseline and follow-up data (N = 48; mean [SD] follow-up of 4.9 [3.03] years), mean (SD) uKS was 33.5 (20.15) µg/mg creatinine at baseline and decreased to 14.4 (10.89) µg/mg creatinine at last follow-up (*p* < 0.0001; Fig. 1b). Mean (SD) decrease from baseline was 19.13 (13.48) µg/mg creatinine or 56.22 (20.75) %. Analysis by age group at treatment initiation showed that uKS levels were higher in patients under 18 years of age while adult patients often had normal or virtually normal baseline uKS levels (Additional file 1: Figure S2).

#### Weight

Weight remained stable over time after reaching adult height (Additional file 1: Figure S3). Younger patients tended to gain weight in line with growth and then stabilised. Weight data were available for ten patients < 5 years old at baseline. Of these, seven had a baseline weight within normal Centers for Disease Control and Prevention (CDC) norms, while weight was below the 3rd percentile in three patients. Only three patients, who were all 4 years of age at baseline, had follow-up data for more than a year. These patients showed a deviation from normal CDC weight-for-age curves over time. In two of them, weight fell below the 3rd percentile by 8 and 9 years of age, while in the third child weight remained within normal limits up to 10 years of age.

#### Endurance

**Fig. 2:**
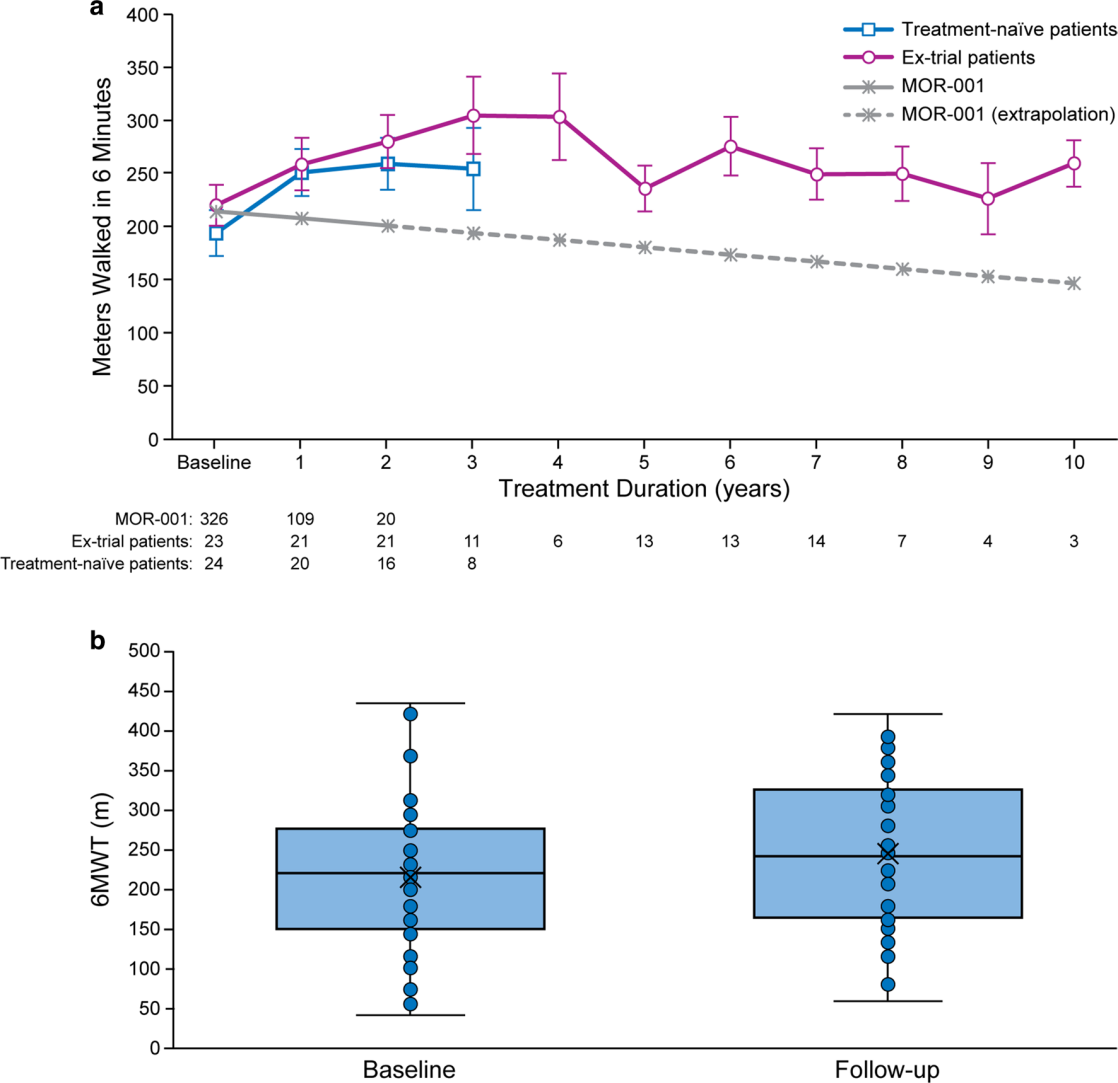
Six-minute walk test (6MWT) distance over time compared to results from untreated patients from the MOR-001 natural history study (MOR-001 data linearly extrapolated beyond year 2) (**a**); box plot of 6MWT at baseline and at last measurement (N = 41; mean follow-up 4.9 years) (**b**). In the box plot, lines show medians and first and third quartiles; asterisks show means

Endurance results showed an initial increase in 6MWT distance and a stabilisation thereafter (Fig. 2a). In patients with both baseline and follow-up data (N = 41), mean (SD) 6MWT distance was 217.05 (97.50) m at baseline and increased to 243.92 (89.19) m at last follow-up (*p* = 0.136; Fig. 2b). Mean (SD) follow-up duration for these patients was 4.9 (2.97) years. Mean (SD) change from baseline was 26.88 (73.33) m.

The majority of ex-trial patients demonstrated long-term 6MWT distance stability with a mean (SD) increase of 12.82 (82.96) m or 14.10 (54.67) % from baseline to last follow-up (N = 21; mean [SD] follow-up of 7.6 [1.45] years). For patients initiating treatment in the MAA, mean (SD) 6MWT distance increased 41.46 (60.25) m or 45.80 (74.87) % from baseline to last follow-up (N = 20; mean [SD] follow-up of 2.16 [0.64] years). Both treatment-naïve and ex-trial patients had better outcomes than would have been expected without treatment, based on the MOR-001 natural history study data (Fig. 2a).

An additional analysis of patients previously enrolled in MOR-002 revealed prolonged durability of treatment effect (Additional file 1: Figure S4). Despite the progressive nature of the disease, mean (SD) percent change from baseline in 6MWT distance was still 9.78 (23.47) % above baseline levels in this group after a mean follow-up of 9.4 (0.45) years (N = 8).

Analysis by age at treatment initiation revealed that patients initiating treatment before 18 years of age had higher 6MWT results than those initiating treatment at or after 18 years of age (Additional file 1: Figure S5). However, the biggest improvements from baseline were seen in the older patients, while those initiating treatment earlier tended to show stable 6MWT results over time.

#### Pulmonary function

**Fig. 3:**
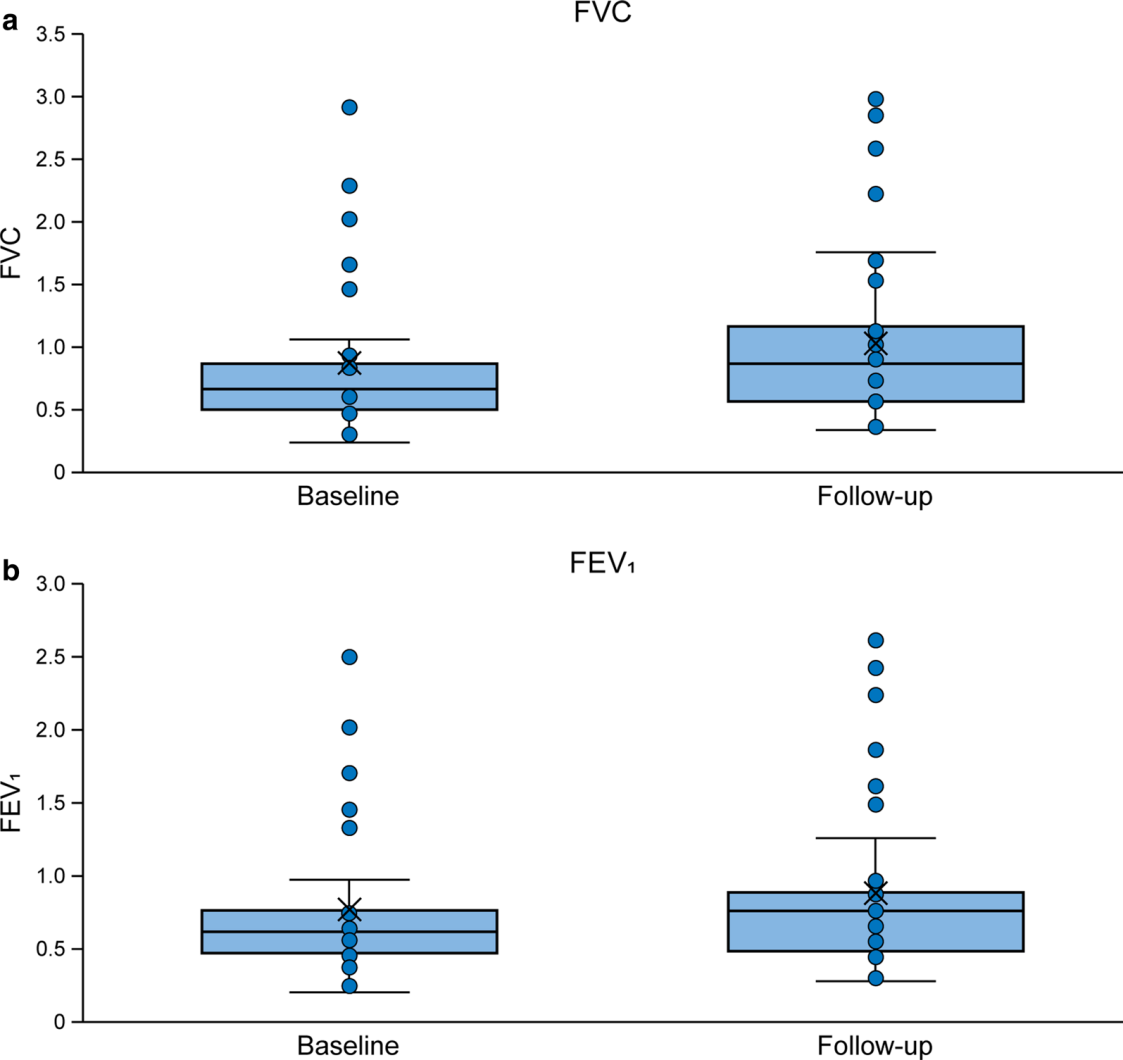
Box plots of pulmonary function (FVC [**a**] and FEV_1_ [**b**]) at baseline versus last follow-up in all patients (N = 40; mean follow-up 5.5 years). Lines show medians and first and third quartiles; asterisks show means

FVC and FEV_1_ were stable or improved numerically over time with treatment (Fig. 3). In patients with both baseline and follow-up data (N = 40), mean (SD) FVC changed from 0.87 (0.61) L at baseline to 1.05 (0.67) L at the last measurement (*p* = 0.216; mean follow-up 5.5 [2.92] years); mean (SD) percent change from baseline to the last measurement was 16.14 (36.04) %. Mean (SD) FEV_1_ changed from 0.78 (0.52) L at baseline to 0.88 (0.58) L at the last measurement (*p* = 0.407; mean follow-up 5.5 [2.92] years); mean percent change from baseline was 15.59 (30.01) %. In contrast with natural history data, lung function was stable or improved regardless of age at treatment initiation (Additional file 1: Figure S6). Overall, approximately half of the patients showed an improvement in pulmonary function (increase of ≥ 0.1 L in FVC and/or FEV_1_); over 85% showed an improvement or stabilisation (Additional file 1: Figure S7).

#### Left ventricular ejection fraction (LVEF)

Mean (SD) LVEF was 65.7 (8.2) % at baseline (mostly measured after treatment initiation) and 66.1 (6.1) % at last follow-up (N = 43). All patients had a LVEF within the normal range at last follow-up.

### Patient-reported outcomes (PROs)

#### Activities of daily living

**Fig. 4:**
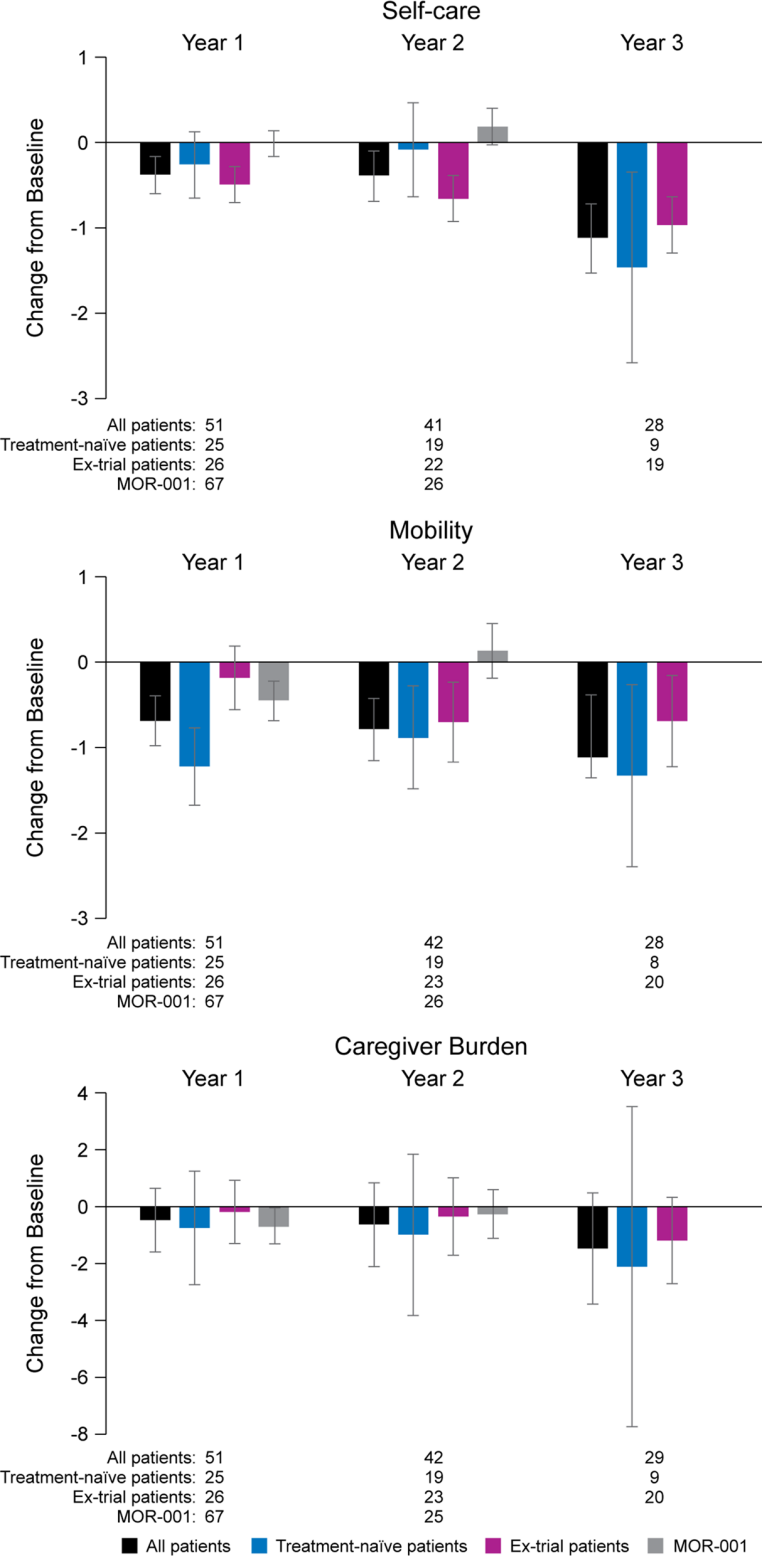
Change from baseline in MPS-HAQ domains over years 1, 2 and 3 of the MAA compared to change from baseline over 1 and 2 years reported in the MOR-001 natural history study. For ex-trial patients, pre-treatment baseline was used. Reductions in MPS-HAQ scores represent improvements. Error bars represent standard error

MPS-HAQ data showed numerical improvements (i.e. decreases) across all domains over 3 years (Fig. 4). These improvements were mainly driven by improvements in the patients starting treatment in the MAA. However, patients who started treatment in the clinical trials and had been on treatment long-term also showed mean improvements across all domains. In patients with both baseline and follow-up data, mean (SD) Caregiver burden score changed from 5.72 (2.75) at baseline to 4.92 (2.96) at last follow-up (N = 38; mean follow-up 5.75 [2.83] years). Mean (SD) Self-care and Mobility scores in these patients were 32.21 (11.51) and 6.59 (2.37), respectively at baseline and 30.5 (11.63) and 5.72 (2.87) at last follow-up.

As a comparison, MPS-HAQ data from untreated patients in the MOR-001 natural history study showed least-square mean (SD) changes from baseline over 2 years in the Caregiver-assistance, Mobility and Self-care domains of -0.5 (0.8), 0.3 (0.3) and 0.4 (0.2) points, respectively.

Wheelchair status remained unchanged in most patients (79%; N = 38). Figure S8 (Additional file 1) provides more details on patients showing stability, decline, or improvement in wheelchair status over time in the MAA and in the MOR-001 natural history study.

#### Other patient-reported outcomes (PROs)

QoL (the EQ-5D-5L utility score) remained stable over time; patients initiating treatment in the MAA showed improvements (Additional file 1: Figure S9).

Pain severity scores remained relatively stable over time. Improvements over time in pain severity were mainly seen in younger patients (completing the APPT), while adults (completing the BPI) showed no substantial change from a mean (SD) baseline score of 1.14 (1.74) (Additional file 1: Figure S10). Mean baseline BDI score was below 13 (mean [SD] = 4.90 [4.80]), indicating no depression, and remained below this threshold during follow-up (Additional file 1: Figure S11). There were no substantial changes in mean BDI scores over time, with SDs exceeding mean changes over baseline in both ex-trial patients and patients who started treatment in the MAA at any time point.

Of note, these assessments were first performed at enrolment in the MAA in all subjects, which means that subjects previously enrolled in clinical trials had been on treatment for several years at the time of baseline assessment. This may explain the relatively good baseline scores and stable follow-up scores in most of these patients.

### Safety

No patients in the MAA stopped treatment due to adverse reactions and antibody titres were in line with previously published reports [13, 24]. For the latest published safety information on the global population, the label should be consulted [10].

## Discussion

Overall, the real-world MAA data are consistent with the results as reported for the clinical trials [11, 13, 14, 17, 18]. In line with international guidelines, elosulfase alfa alongside multidisciplinary care can positively impact clinical and PRO measures. Patients enrolled in the programme showed a rapid decrease and a subsequent stabilisation over the long term in uKS, as well as initial improvements in endurance and pulmonary function and then stabilisation in these measures in the long term. In addition, patients’ ability to perform ADL and the need for caregiver assistance improved upon treatment initiation and remained stable in patients on long-term treatment. Trends towards improvement or stabilisation of these outcomes were seen regardless of age at which treatment was started (before or after 18 years of age).

The finding that almost all patients in the MAA remained on therapy during the study period further confirms clinical stability of these patients, since they were only allowed to continue treatment if they met four out of five MAA clinical and PRO criteria outlined in Table 1.

In patients who started therapy in the MAA, mean changes from pre-treatment baseline in 6MWT distance (+ 41 m) and uKS (− 56%) were in accordance with those reported in the clinical trials. In patients from the MOR-004/005 Intent-To-Treat population continuously treated with elosulfase alfa 2.0 mg/kg/week, least square mean changes from baseline after 2 years were + 32 m for 6MWT distance and − 64% for uKS [13]. It is important to be aware that the MAA included a significant number of patients who would not have qualified for the pivotal trials, i.e. children under 5 years of age and adults with non-classical disease that have relatively good 6MWT results at baseline. In the phase 3 clinical trials, only patients ≥ 5 years and with a 6MWT distance ≥ 30 to ≤ 325 m were enrolled. As the MAA population is not as uniform, the mean change in 6MWT, for example, can be considered a less representative measure than it was in the MOR-004/005 trial. This makes it all the more remarkable that most patients met the MAA criteria for continuing treatment.

Patients who were previously enrolled in clinical trials and had been on long-term therapy (mean [SD] treatment duration of 7.46 [1.62] years) showed stability of endurance and pulmonary function, as opposed to the progressive decline that would be expected based on the natural history of MPS IVA. A separate analysis of the patients with the longest duration of treatment (MOR-002 patients; mean [SD] treatment duration of 9.4 [0.45] years) further confirmed the positive impact of treatment on endurance over an extended period of time. As all patients in the MAA received ERT, outcomes could not be directly compared with a control group of untreated patients to assess the impact of treatment on the disease course. Therefore, 6MWT results were compared with results obtained from patients in the MOR-001 natural history study. Untreated MOR-001 patients showed a clear deterioration in 6MWT distance over time, while mean 6MWT results remained above baseline levels for MAA patients throughout the study period, regardless of duration of treatment or age at treatment initiation. In addition, MAA patients showed better pulmonary function outcomes over 3 years than the MOR-001 population over 2 years. MPS-HAQ results in the MAA were also more favourable than those reported for the MOR-001 population, which showed a deterioration in Mobility and Self-care over a 2-year time period [17]. Overall, these results suggest that ERT can slow down the gradual regression in endurance and function associated with MPS IVA. It should be noted that differences in patient monitoring and supportive care may also have contributed to the different outcomes in the MAA and MOR-001 populations.

A limitation of the analysis is that pre-treatment baseline data were not available for all patients, due to a young age at treatment initiation or an endpoint not being measured at baseline for ex-trial patients. PROs, with exception of the MPS-HAQ, were not measured before treatment initiation in ex-trial patients. In addition, most patients (both ex-trial and treatment-naïve patients) had no pre-treatment baseline for LVEF. In these cases, the first measure during the MAA period had to be used as baseline. However, for most endpoints, only a minority of the total study population had no pre-treatment baseline. Moreover, the use of baseline measures collected after treatment initiation may have resulted in an underestimation, rather than an overestimation, of treatment effects. Finally, some of the follow-up data during the programme were missing due to subjects being unable to complete the test at the time of the measurement (due to age, surgery, illness, or missing the follow-up appointment), or a delay in data availability. This led to lower patient numbers in some of the analyses, including pain and BDI evaluations. PRO tools used in the study are also not validated for MPS IVA and may therefore not be sensitive to all the issues associated with this progressive disease.

It should be emphasised that none of the measures evaluated in the MAA can be considered an accurate reflection of the benefits of treatment on its own. Due to the wide phenotypic heterogeneity of MPS IVA patients, it is important to look at different measures in concert, supporting a holistic approach for monitoring these patients. The measures chosen as part of the monitoring in the MAA should also not be viewed as the only important measures for monitoring patients’ health. Other measures, such as skeletal and joint abnormalities, impairments in vision and hearing, and sleep problems, are also important in the regular follow-up of these patients, to allow timely interventions (such as surgeries) and optimal patient outcomes. The newly published international recommendations for the management of MPS IVA provide a detailed list of assessments recommended for these patients [9].

## Conclusions

The real-world, long-term results of the MAA are meaningful for patients with MPS IVA, who typically experience early morbidity and mortality without proper treatment. Overall, the presented data provide further evidence that long-term treatment with elosulfase alfa slows down the progressive deterioration in endurance associated with the disease, has a positive impact on pulmonary function and patients’ ability to perform ADL and lessens their need for caregiver assistance. While ERT is not expected to result in normalisation of clinical parameters, appropriate continued therapy leads to clinically meaningful improvements in some parameters and a slower progression of this progressive debilitating disease overall. Continued collection and analysis of real-world efficacy and safety data of patients treated with elosulfase alfa will help to better understand the long-term effects of this therapy. This is particularly important for patient subgroups who were not included in the clinical trials, such as the very youngest patients for whom early intervention to potentially prevent irreversible damage is of particular importance.

## Supplementary Information

**Additional file 1:** **Table S1.** Exclusion and starting criteria of the MAA. **Table S2.** Overview of assessments in the MAA. **Figure S1.** Flow chart of patient disposition. **Figure S2.** Urinary keratan sulfate (uKS) over time by age at treatment initiation. Error bars are standard error. **Figure S3.** Weight over time by age at treatment initiation. Error bars are standard error. **Figure S4.** 6-minute walk test (6MWT) distance outcomes over time for patients from MOR-002 compared to natural history data from MOR-001 (MOR-001 data linearly extrapolated beyond year 2). Error bars are standard error. **Figure S5.** 6-minute walk test (6MWT) distance over time by age at treatment initiation compared to results from untreated patients from the MOR-001 natural history study (MOR-001 data linearly extrapolated beyond year 2). Error bars are standard error. **Figure S6.** Change in FVC (A) and FEV1 (B) over time by age group with comparison to MOR-001 natural history (MOR-001 data available for FVC only, linearly extrapolated beyond year 2). Error bars are standard error. **Figure S7.** Changes in pulmonary function from baseline to last follow-up (N = 40). Decline: ≥ 0.1 L decrease; Improvement: ≥ 0.1 L increase; Stability: < 0.1 L increase or decrease. **Figure S8.** Patients showing stability, decline, or improvement in wheelchair status over time versus baseline (based on MPS-HAQ Mobility Q33 and Q33a regarding wheelchair use); all MAA patients combined (N = 38; mean [SD] follow-up of 5.75 [2.83] years) are compared to MOR-001 natural history subjects (N = 73; mean [SD] follow-up of 2.32 [1.06] years). Decline: change from no use at baseline to some/always use at last follow-up, or from some use at baseline to always use at last follow-up; Improvement: change from some/always use at baseline to no use at last follow-up, or from always use at baseline to some use at follow-up; Stability: no change in status from baseline to last follow-up. **Figure S9.** Change from baseline in EQ-5D-5L utility score over time in all patients and by trial history. Increasing scores represent improvements in quality of life. Error bars are standard error. **Figure S10.** Pain severity as assessed with the Adolescent Paediatric Pain Tool (APPT; patients aged <18 years) (A) and Brief Pain Inventory (BPI; patients aged ≥18 years) (B) over time by trial history. Decreasing scores represent improvements. Error bars are standard error. **Figure S11.** Beck Depression Inventory (BDI) score* change from baseline over time. Decreasing scores represent improvements. Error bars are standard error.

## Data Availability

The de-identified individual participant data that underlie the results reported in this article (including text, tables, figures, and appendices) will be made available together with the research protocol and data dictionaries, for non-commercial, academic purposes. Additional supporting documents may be available upon request. Investigators will be able to request access to these data and supporting documents via a website (www.BioMarin.com) beginning 6 months and ending 2 years after publication. Data associated with any ongoing development program will be made available within 6 months after approval of relevant product. Requests must include a research proposal clarifying how the data will be used, including proposed analysis methodology. Research proposals will be evaluated relative to publicly available criteria available at www.BioMarin.com to determine if access will be given, contingent upon execution of a data access agreement with BioMarin Pharmaceutical Inc.
