# Supplementary material for: Impact of long-term elosulfase alfa treatment on clinical and patient-reported outcomes in patients with mucopolysaccharidosis type IVA: results from a Managed Access Agreement in England
Source: Orphanet J Rare Dis. 2021 Jan 21;16:38. doi: 10.1186/s13023-021-01675-x (PMC7818902; doi:10.1186/s13023-021-01675-x)
Supplement: Supplementary file 1 — Additional file 1: Table S1. Exclusion and starting criteria of the MAA. Table S2. Overview of assessments in the MAA. Figure S1. Flow chart of patient disposition. Figure S2. Urinary keratan sulfate (uKS) over time by age at treatment initiation. Error bars are standard error. Figure S3. Weight over time by age at treatment initiation. Error bars are standard error. Figure S4. 6-minute walk test (6MWT) distance outcomes over time for patients from MOR-002 compared to natural history data from MOR-001 (MOR-001 data linearly extrapolated beyond year 2). Error bars are standard error. Figure S5. 6-minute walk test (6MWT) distance over time by age at treatment initiation compared to results from untreated patients from the MOR-001 natural history study (MOR-001 data linearly extrapolated beyond year 2). Error bars are standard error. Figure S6. Change in FVC (A) and FEV1 (B) over time by age group with comparison to MOR-001 natural history (MOR-001 data available for FVC only, linearly extrapolated beyond year 2). Error bars are standard error. Figure S7. Changes in pulmonary function from baseline to last follow-up (N = 40). Decline: ≥ 0.1 L decrease; Improvement: ≥ 0.1 L increase; Stability: < 0.1 L increase or decrease. Figure S8. Patients showing stability, decline, or improvement in wheelchair status over time versus baseline (based on MPS-HAQ Mobility Q33 and Q33a regarding wheelchair use); all MAA patients combined (N = 38; mean [SD] follow-up of 5.75 [2.83] years) are compared to MOR-001 natural history subjects (N = 73; mean [SD] follow-up of 2.32 [1.06] years). Decline: change from no use at baseline to some/always use at last follow-up, or from some use at baseline to always use at last follow-up; Improvement: change from some/always use at baseline to no use at last follow-up, or from always use at baseline to some use at follow-up; Stability: no change in status from baseline to last follow-up. Figure S9. Change from baseline in EQ-5D-5L utility score over [file 13023_2021_1675_MOESM1_ESM.docx]

**Additional file 1**

**Table S1.** Exclusion and starting criteria of the MAA

| **Elosulfase alfa will not be started if any of the following apply:**   - The patient is diagnosed with an additional progressive life limiting condition where treatment would not provide long-term benefit (e.g. cancer or multiple sclerosis); or - The patient has a lung capacity (forced vital capacity) of <0.3 L and requires ventilator assistance; or - The patient is unwilling to comply with the associated monitoring criteria:   - All patients are required to attend their clinics three times a year for assessment   - All patients will sign up to the ‘Managed Access Patient Agreement’ (NICE 2019) |
| --- |
| **All of the following are required before treatment is started:**   - All patients must have a confirmed diagnosis of MPS IVA as per the diagnosis criteria recommended in Wood et al. (2012) - All patients must have confirmed enzymatic test, elevated urinary keratan sulfate and mutation analysis - In addition, patients aged ≥5 years can only start once a full set of baseline assessments has been obtained, and they have signed the Managed Access Patient Agreement |

**References:**

National Institute for Health and Care Excellence (NICE). Managed Access Agreement elosulfase alfa for treating mucopolysaccharidosis type IVA. <https://www.nice.org.uk/guidance/hst2/resources/managed-access-agreement-december-2015-pdf-2238935869>. Accessed 12 Dec, 2019.

Wood T, Bodamer OA, Burin MG, et al. Expert recommendations for the laboratory diagnosis of MPS VI. Mol Genet Metab. 2012;106(1):73-82.

**Table S2.** Overview of assessments in the MAA

| **Assessments** | **Baseline** | | **Month 4** | | **Month 8** | | **Month 12** |
| --- | --- | --- | --- | --- | --- | --- | --- |
| 6MWT or T25FW | X | | X | |  | | X |
| FVC | X | |  | | X | |  |
| FEV_1_ | X | |  | | X | |  |
| uKS | X | X | |  | | X | |
| EQ-5D-5L | X | |  | |  | | X |
| MPS-HAQ caregiver | X | |  | |  | | X |
| Beck Depression Score | X | |  | | X | |  |
| BPI/APPT | X | | X | | X | | X |
| Cardiac echo (LVEF) | X | |  | |  | | X |
| Missed infusions |  | | X | | X | | X |
| Weight | X | | X | | X | | X |
| Antibody titres | X | |  | | X | | X |

6MWT: 6-minute walk test; APPT: Adolescent Paediatric Pain Tool; BPI: Brief Pain Inventory; EQ-5D-5L: EuroQol 5 dimensions, 5 levels; FEV_1_: forced expiratory volume in 1 second; FVC: forced vital capacity; LVEF: left ventricular ejection fraction; MPS-HAQ: MPS Health Assessment Questionnaire; T25FW: timed 25-foot walk; uKS: urinary keratan sulfate

**Figure S1.** Flow chart of patient disposition.


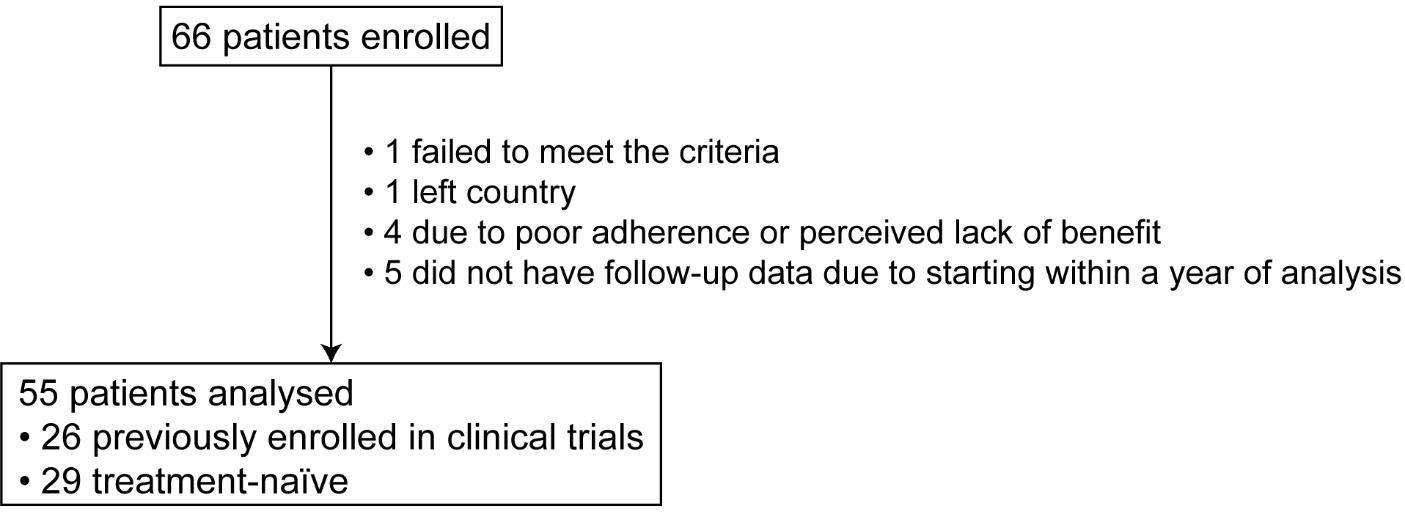


**Figure S2.** Urinary keratan sulfate (uKS) over time by age at treatment initiation. Error bars are standard error.


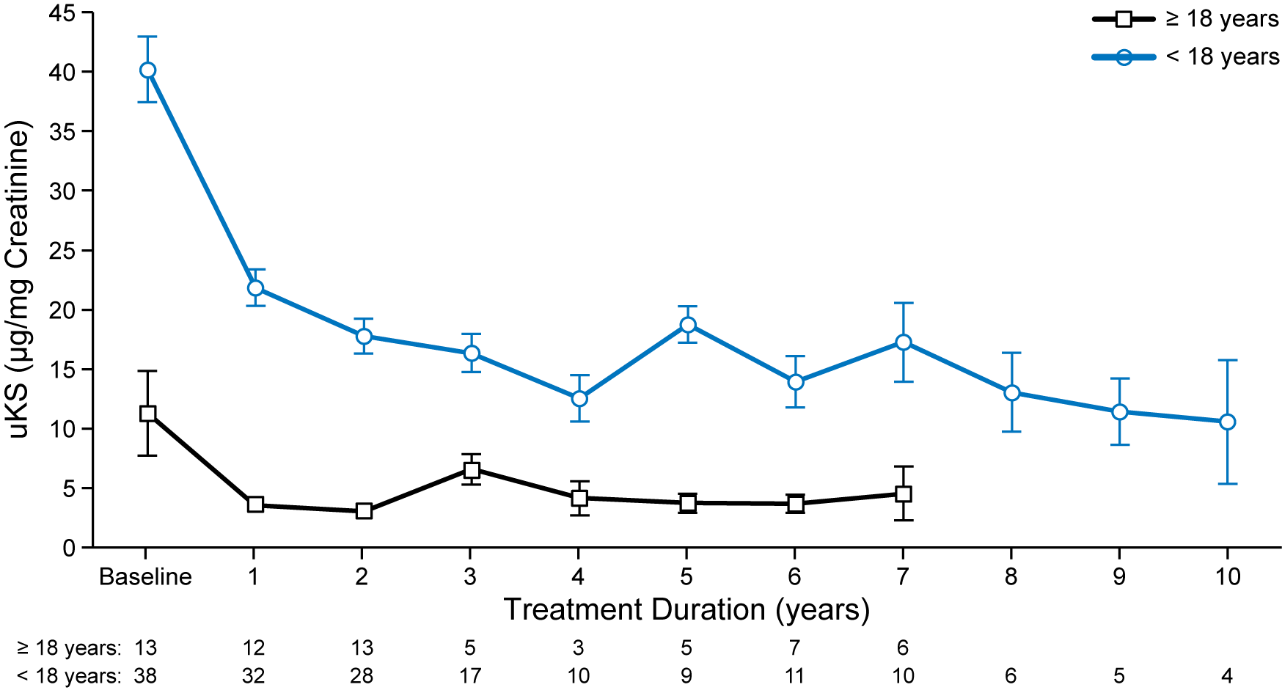


**Figure S3.** Weight over time by age at treatment initiation. Error bars are standard error.


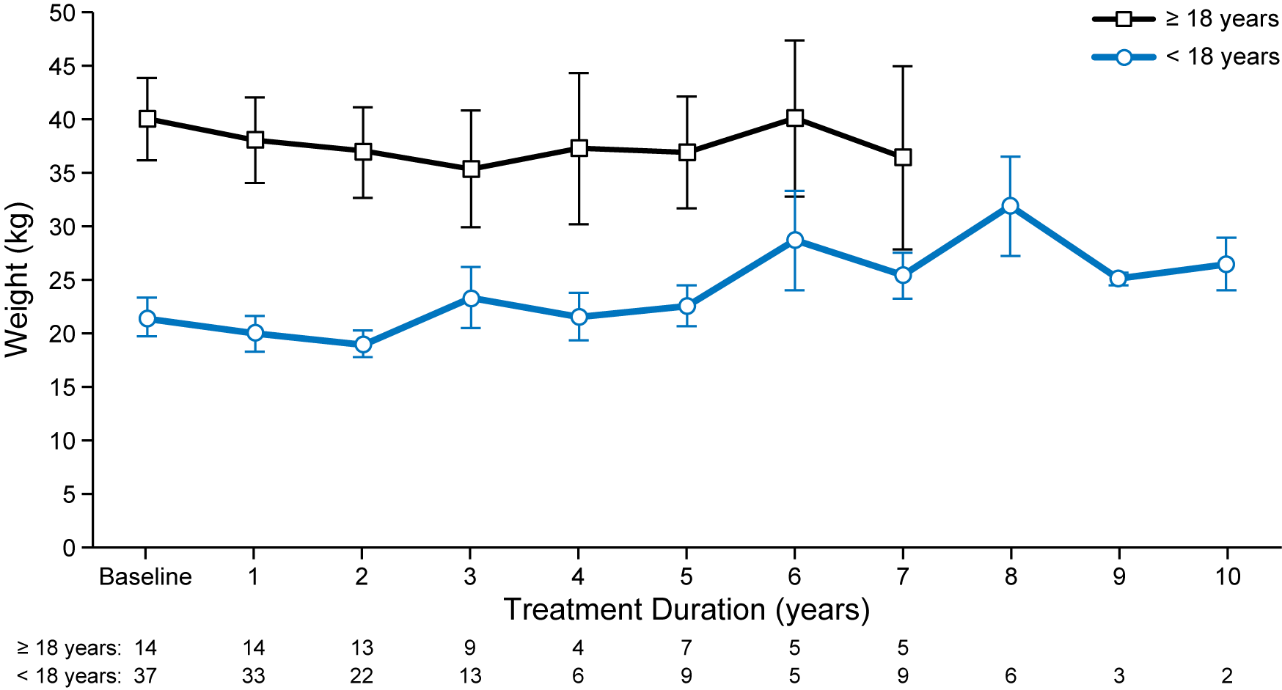


**Figure S4.** 6-minute walk test (6MWT) distance outcomes over time for patients from MOR-002 compared to natural history data from MOR-001 (MOR-001 data linearly extrapolated beyond year 2). Error bars are standard error.


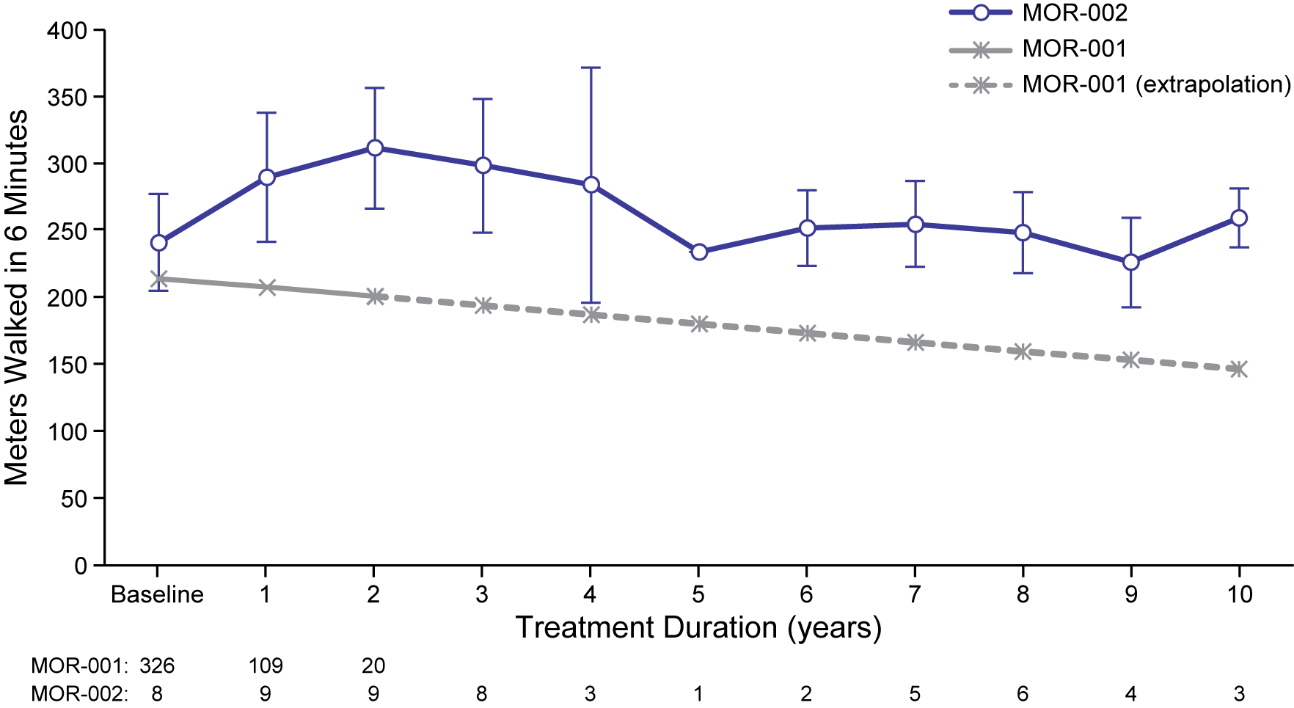


**Figure S5.** 6-minute walk test (6MWT) distance over time by age at treatment initiation compared to results from untreated patients from the MOR-001 natural history study (MOR-001 data linearly extrapolated beyond year 2). Error bars are standard error.

**
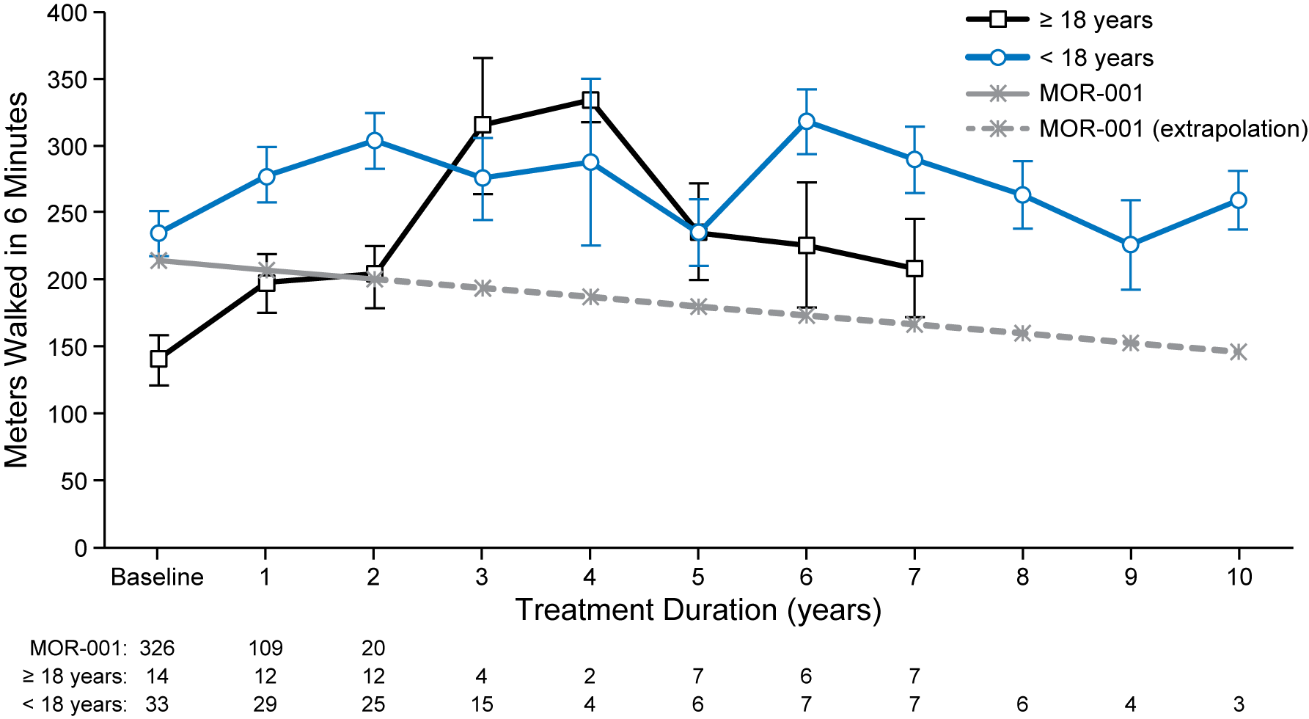
**

**Figure S6.** Change in FVC (**A**) and FEV_1_ (**B**) over time by age group with comparison to MOR-001 natural history (MOR-001 data available for FVC only, linearly extrapolated beyond year 2). Error bars are standard error.


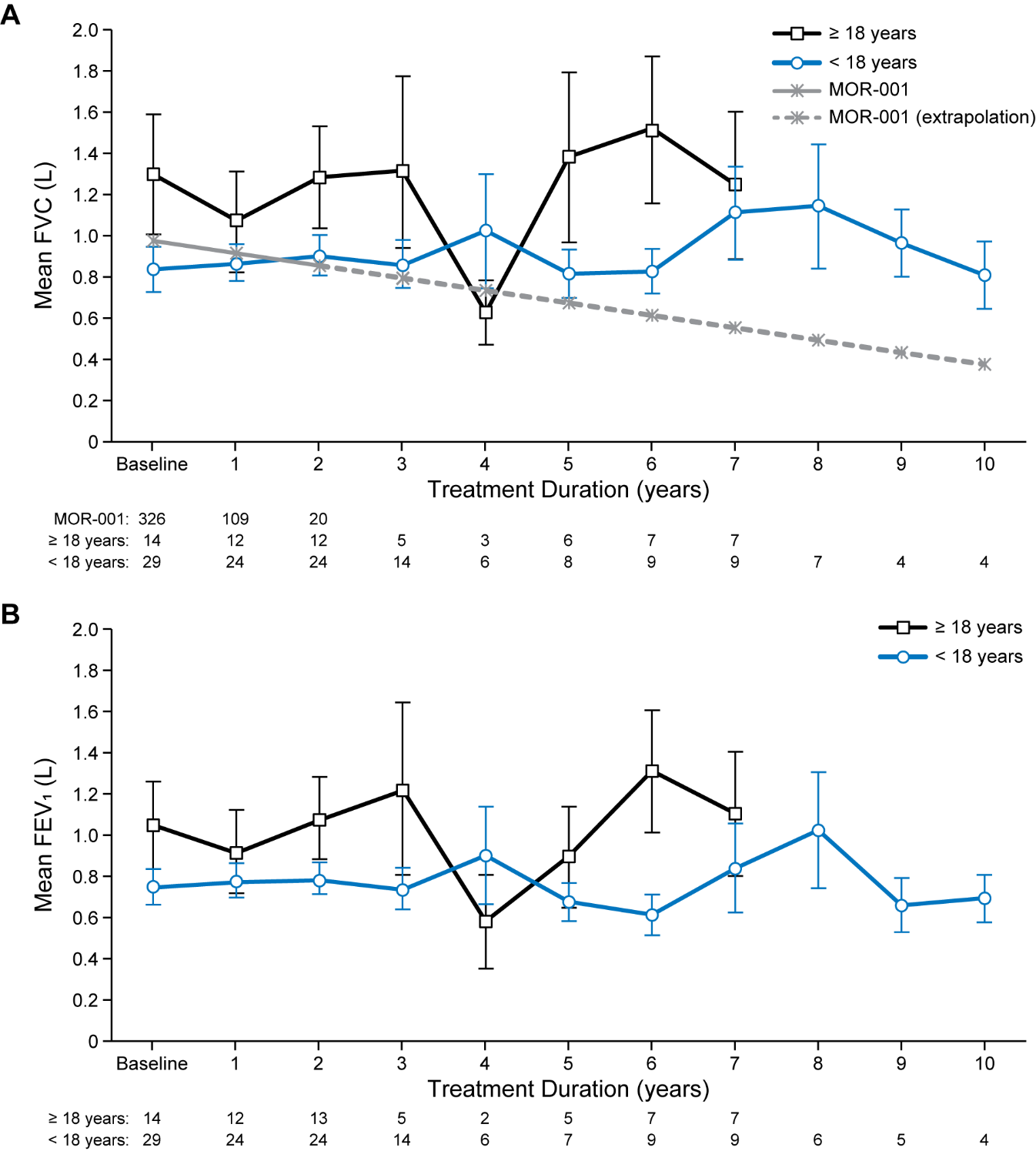


Note: FEV_1_ was not measured in MOR-001

**Figure S7.** Changes in pulmonary function from baseline to last follow-up (N=40). Decline:
≥0.1 L decrease; Improvement: ≥0.1 L increase; Stability: <0.1 L increase or decrease.


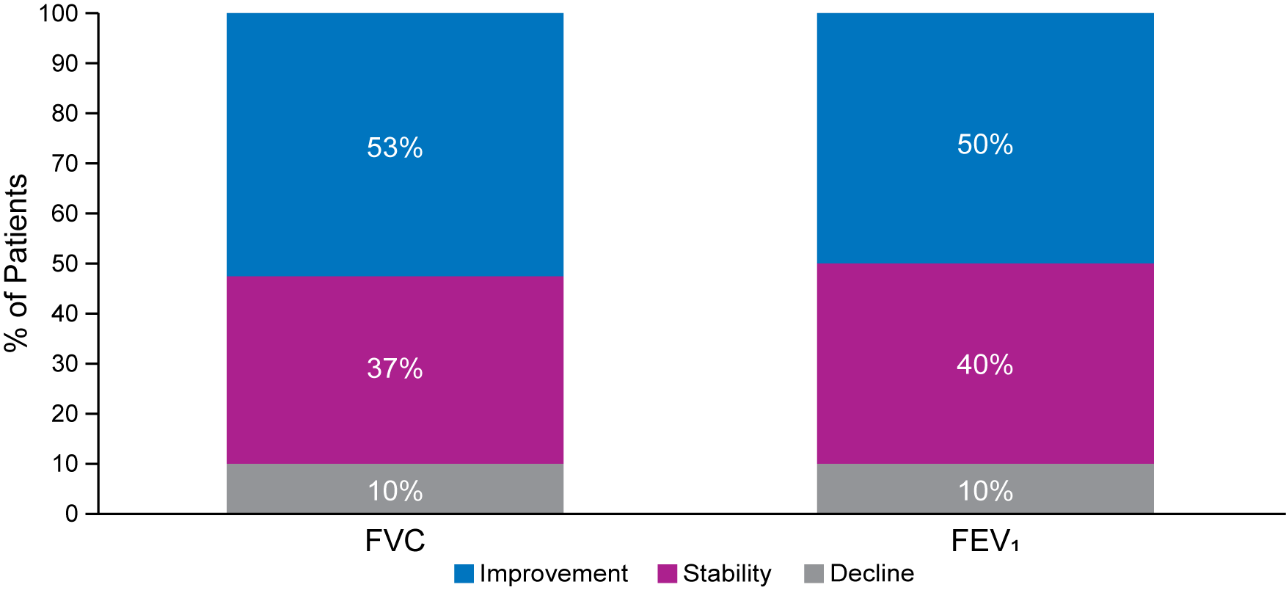


**Figure S8.** Patients showing stability, decline, or improvement in wheelchair status over time versus baseline (based on MPS-HAQ Mobility Q33 and Q33a regarding wheelchair use); all MAA patients combined (N=38; mean [SD] follow-up of 5.75 [2.83] years) are compared to MOR-001 natural history subjects (N=73; mean [SD] follow-up of 2.32 [1.06] years). Decline: change from no use at baseline to some/always use at last follow-up, or from some use at baseline to always use at last follow-up; Improvement: change from some/always use at baseline to no use at last follow-up, or from always use at baseline to some use at follow-up; Stability: no change in status from baseline to last follow-up.

**
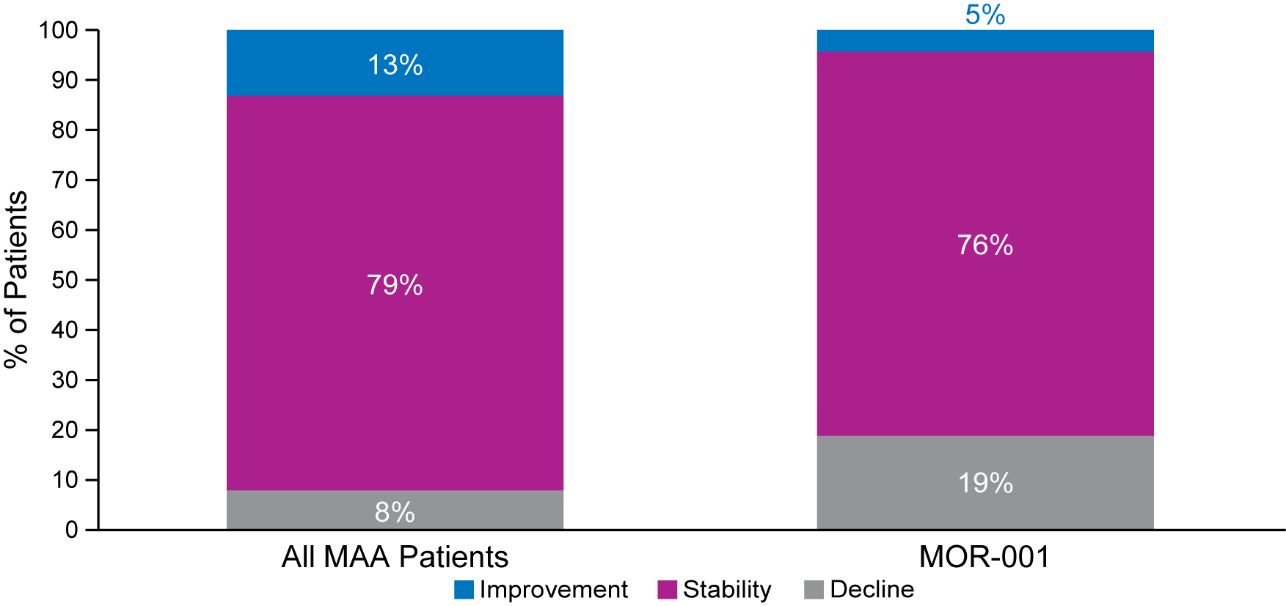
**

**Figure S9.** Change from baseline in EQ-5D-5L utility score over time in all patients and by trial history. Increasing scores represent improvements in quality of life. Error bars are standard error.


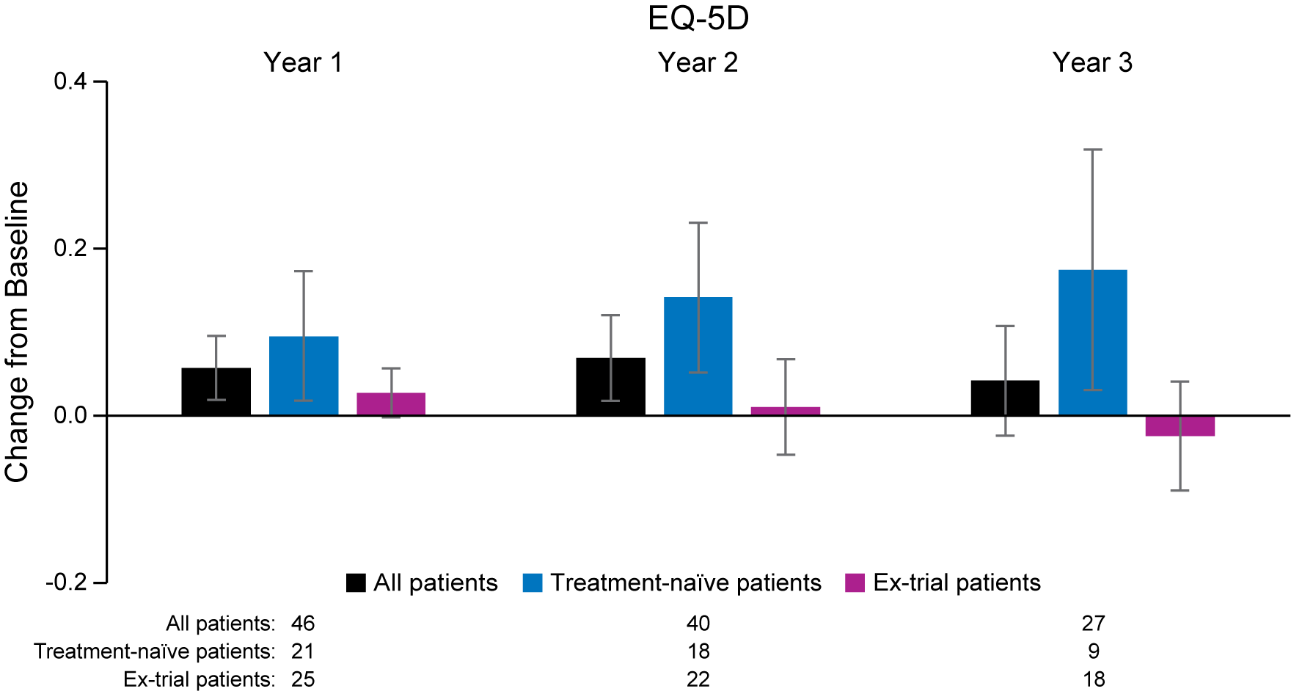


**Figure S10.** Pain severity as assessed with the Adolescent Paediatric Pain Tool (APPT; patients aged <18 years) (**A**) and Brief Pain Inventory (BPI; patients aged ≥18 years) (**B**) over time by trial history. Decreasing scores represent improvements. Error bars are standard error.


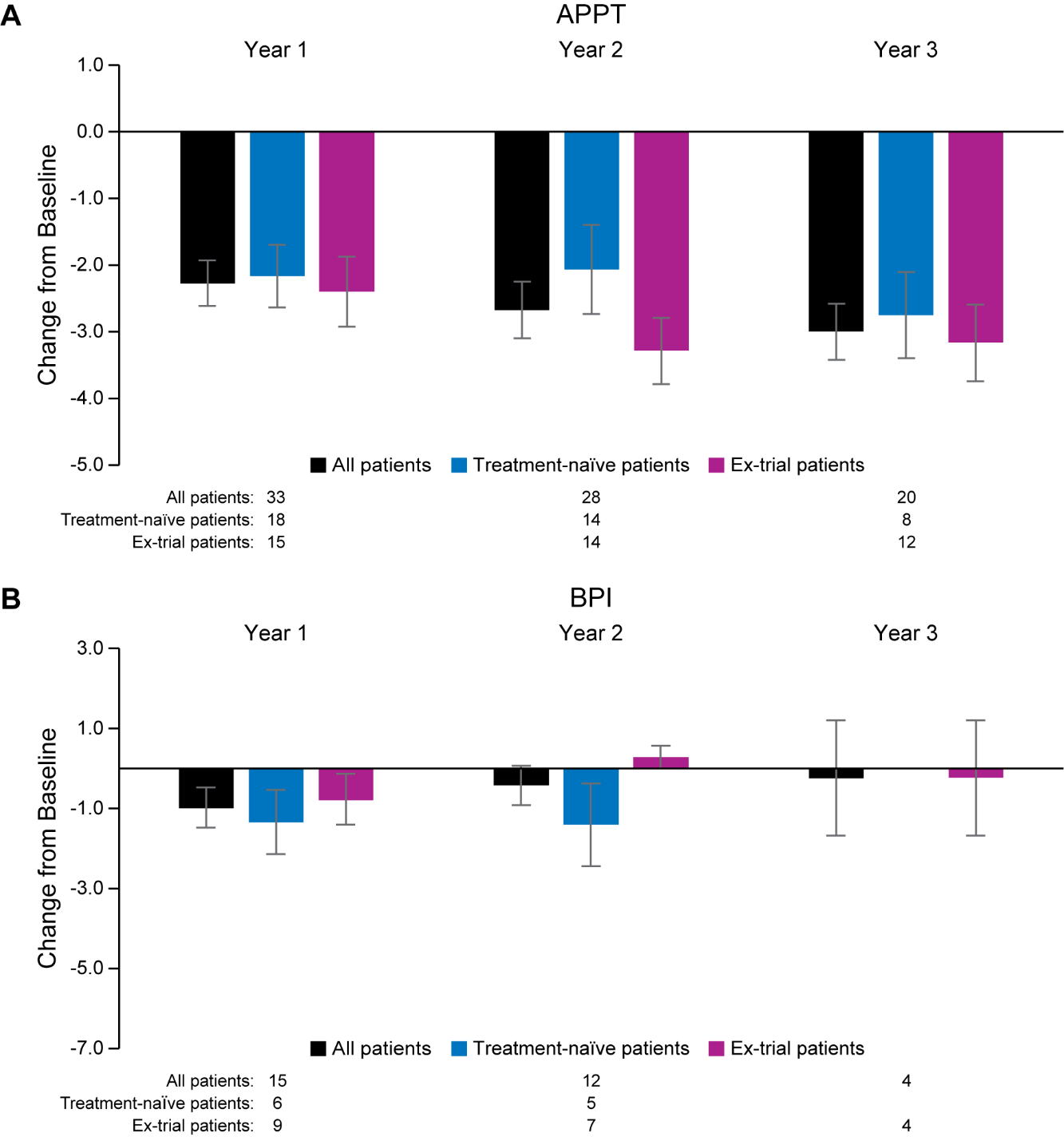


**Figure S11.** Beck Depression Inventory (BDI) score* change from baseline over time. Decreasing scores represent improvements. Error bars are standard error.


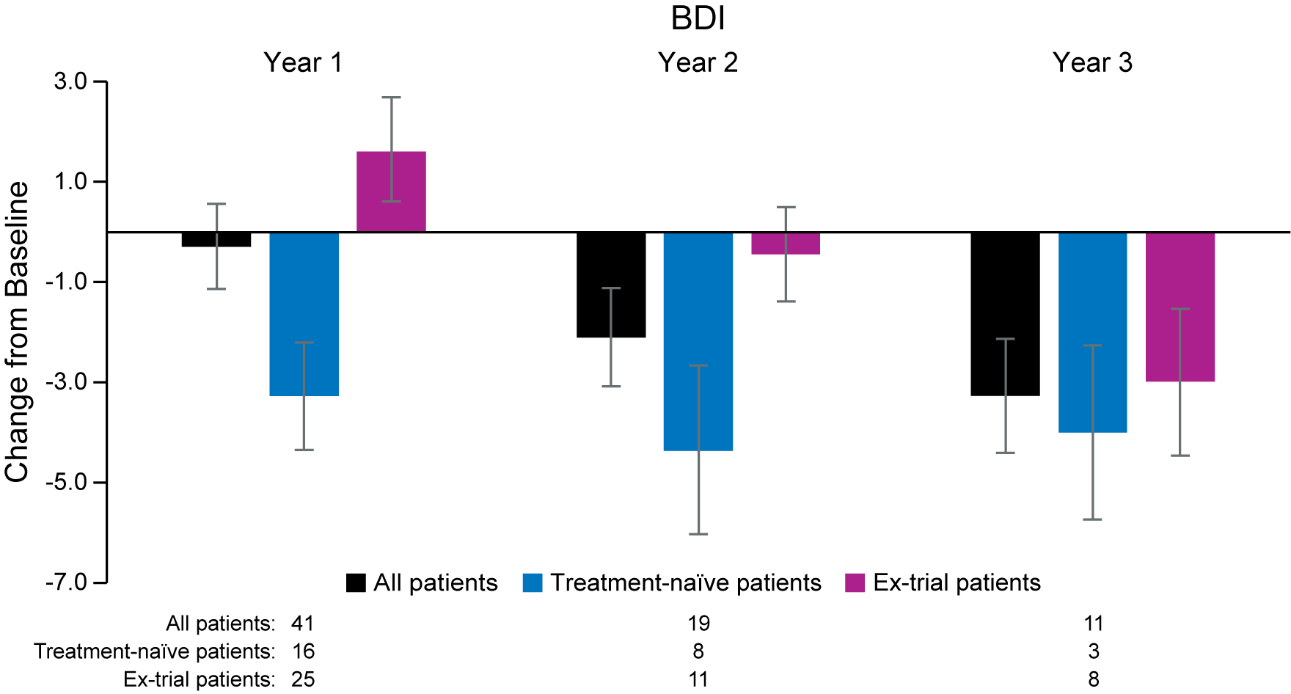


*Beck Depression Inventory was only administered in patients ≥13 years of age
